# Active or passive tense?

**DOI:** 10.1093/jhps/hnab052

**Published:** 2021-08-08

**Authors:** Richard (Ricky) Villar

**Affiliations:** *Editor-in-Chief,* Journal of Hip Preservation Surgery

I find it perplexing that an author can spend months, even years, undertaking a research project, discover globe-shattering results, and then somehow rush their submission. They lose focus when it comes to the quality of their writing. I have lost track of the number of times an author challenges the rejection of their paper by protesting, ‘But I did say such-and-such. The reviewer clearly did not read the manuscript’.

The fact is that it is up to us, as authors, to be sure that reviewers read what we wish them to see. In the wider world of writing for the mass market, and away from academic publishing, writers say to one another that if they are rejected, the best place to look is in the mirror. For academic publishing, if a reviewer misses something, we should blame ourselves, not the reviewer. It is our job to keep the reviewer focused. To make this possible, our use of English, especially for an English-language journal such as *JHPS*, must be immaculate. That is not always easy for a global journal.

Since the arrival of an impact factor for *JHPS*, so our submission rate has rocketed, with wonderful papers from right around the world. Yet not every country uses English as its first language. If you count yourself among this group, please think about the use of an editing agency before you submit your paper.

It is also helpful to understand how a reader reads. For example, a typical reading speed is 250 words/min, with readers and dare I say it reviewers, rarely digesting a full paper. If you can persuade a reader to look at more than 20% of your writing, you are doing well. For a reviewer, I would estimate this might rise to 60%. It is very uncommon for every word to be digested by those who read your work. Readers will spend 70% of their time on the left-hand side of the page and 30% on the right. Many will scan or skim as they read. Scanning is when you flash through a document to look for a specific word or item. You know what you wish to find but have no idea where it is. Skimming is when you read a paper quickly to obtain a general idea of the meaning. Skimming is very common for scientific readers.

To help a reader focus, there are key points in a submission that are critical. First, the title must be short, but not too short, nor too long, but about right. It needs to carry the nature of the research and perhaps an inkling of the result. Think of when you are undertaking a literature analysis for your research. I wager you will stop for a moment at a paper with an interesting title.

The abstract is key. This is the portion of your paper that the world will see for the rest of time. It is the one part of a paper that anyone can access for free, whether it has been published by a subscription journal or, as with *JHPS* and so many others, Open Access. The first few lines of every section of a paper must be perfectly phrased and spelled, as should the final few lines of each section, too. This is to cater for the skim reader, who will read the beginning of a section slowly, speed up through the body of the text, and slow down toward the end. Think carefully about your use of images and tables. They must not be hard to understand and not so large that the journal needs to publish them sideways. They should not repeat what is already said in the text.

There are then the active and passive tenses with which I admit to an unreasonable obsession. If there was ever a dilemma for a scientific author, this is it. Traditionally, scientific writing has been in the third person, the so-called passive voice. Try these:

‘An acetabular labral tear was seen at the two o’clock position’. This is the passive tense.

As opposed to,

‘We saw an acetabular labral tear at the two o’clock position’. This is the active tense.

Which do you prefer? The active tense has more spirit to it, is more direct, and is something we actually did. No one else. Us. We did it and there is no denying the event. The passive tense is less definite, almost as if the labral tear was seen by chance, perhaps observed by a strange being who may or may not have been an author. If I write a paper, I am proud of the fact, am confident of my results, and want the reader to know it. The active tense is for me.

Next time you write a paper, please think about your tenses, and have a go at the active, rather than passive forms. I wager you will like it. Once active, always active. If passive, all I would ask is why?

Turning to our journal, this journal, *JHPS*, the last issue, number 7.3, was once again a collection of brilliance. All its papers were first rate but two stand out, purely as a reflection of my own interests. One was the paper by Onggo *et al*. [1] on the wave sign. I have lost track of the number of wave signs I have seen over the years. I recognize it immediately when I see it but realize that Onggo *et al*. are correct when they say that this is an operative finding for which plenty of research is still needed to establish the sign’s significance. The other paper was that by Kumar *et al*. [2], who looked at the role of orthobiologic adjuvants when undertaking a core decompression for hip preservation in avascular necrosis of the hip. It appears that the addition of bone marrow aspirate concentrate to a core decompression enhances the efficacy of the procedure.

As for this issue, number 7.4, which is again hopelessly late, I was especially fascinated by two papers. One was the paper by Wickman *et al*. [3] on implementing video visits into an orthopedic hip arthroscopy practice. High-quality care is maintained, as is patient satisfaction, and I suspect we will see more about video-orthopedics as this pandemic era progresses. It is an area on which we should all pay close attention. The other was the physiotherapy consensus document published by Takla *et al*. [4]. This document was prepared using a modified Delphi technique and looks at hip assessment, non-surgical physiotherapy management, prehabilitation, post-operative physiotherapy rehabilitation with its various stages, and return to sports afterwards. I feel sure this document will be used right around the world by many practitioners for a long time to come. Its arrival is perfectly timed and very welcome.

So, as ever, please enjoy this issue of *JHPS*. It is published for you, the hip preservation practitioner, and is filled from cover to cover with brilliance. I commend this issue to you in its entirety.

Oh yes, and please read, use and cite this journal at every opportunity. Ask everyone you know to do the same.

My very best wishes to you all.
